# MiR-21 Is Remotely Governed by the Commensal Bacteria and Impairs Anti-TB Immunity by Down-Regulating IFN-γ

**DOI:** 10.3389/fmicb.2020.512581

**Published:** 2021-01-21

**Authors:** Fang Yang, Yi Yang, Yiwei Chen, Guobao Li, Guoliang Zhang, Lingming Chen, Zhiyi Zhang, Qiongdan Mai, Gucheng Zeng

**Affiliations:** ^1^Department of Microbiology, Zhongshan School of Medicine, Key Laboratory for Tropical Diseases Control of the Ministryof Education, Sun Yat-sen University, Guangzhou, China; ^2^Department of Tuberculosis, Shenzhen Third People’s Hospital, Southern University of Science and Technology, Shenzhen, China; ^3^National Clinical Research Center for Tuberculosis, Guangdong Key Laboratory for Emerging Infectious Diseases, Shenzhen Third People’s Hospital, Southern University of Science and Technology, Shenzhen, China

**Keywords:** commensal bacteria, miRNA, **t**uberculosis, IFN-γ, *Mycobacterium tuberculosis*

## Abstract

Tuberculosis (TB), which is a frequent and important infectious disease caused by *Mycobacterium tuberculosis*, has resulted in an extremely high burden of morbidity and mortality. The importance of intestinal dysbacteriosis in regulating host immunity has been implicated in TB, and accumulating evidence suggests that microRNAs (miRNAs) might act as a key mediator in maintaining intestinal homeostasis through signaling networks. However, the involvement of miRNA in gut microbiota, TB and the host immune system remains unknown. Here we showed that intestinal dysbacteriosis increases the susceptibility to TB and remotely increased the expression of miR-21 in lung. Systemic antagonism of miR-21 enhanced IFN-γ production and further conferred immune protection against TB. Molecular experiments further indicated that miR-21a-3p could specifically target IFN-γ mRNA. These findings revealed regulatory pathways implicating intestinal dysbacteriosis induced-susceptibility to TB: intestinal dysbiosis→lung miRNA→targeting IFN-γ→impaired anti-TB immunity. This study also suggested that deregulated miRNAs by commensal bacteria could become promising targets as TB therapeutics.

## Introduction

*Mycobacterium tuberculosis* (*M. tuberculosis*), the causative agent of tuberculosis (TB), infects one-third of the world population and leads to high morbidity and mortality (Dickson et al., 2016). Clinical options available to combat TB include chemotherapeutic agents and the preventative vaccine *Mycobacterium* Bovis bacillus Calmette–Guérin (BCG) which has been undermined due to the broad emergence of drug-resistant strains of *M. tuberculosis* (Dheda et al., 2014), insufficient protection of BCG efficacy against pulmonary TB in adolescents and adults (Colditz et al., 1994) as well as HIV-1 co-infection. Developing new strategies against tuberculosis such as a better vaccine or a host-directed therapy can be aided by the increasing understanding of the immune responses against *M. tuberculosis* infection (Kaufmann, 2006; Bhatt and Salgame, 2007; Kaufmann et al., 2010).

Microbial community inhabits the skin, the oral, pulmonary, urogenital and gastrointestinal (GI) tract, with the GI tract having the highest density of microorganisms. The gut microbiota plays a vital role in shaping and modulating systemic immune response (Kosiewicz et al., 2011; Schirmer et al., 2016). Given the diverse functional repertoire of the gut microbiota, it is not surprising that several human disorders were reported to be associated with dysbiosis of the resident microbiota (Iida et al., 2013; Tang et al., 2013, 2017; Vetizou et al., 2015; Paun et al., 2016; Sampson et al., 2016). Moreover, accumulating evidence indicates that gut microbiota dysbiosis also provokes susceptibility to infectious diseases. It advocates the importance of commensal bacteria to host health as commensal microbes can calibrate innate and adaptive immune responses and impact the activation threshold for pathogenic stimulations (Ivanov and Honda, 2012). For example, treatment with broad-spectrum antibiotics decreased TNF-α and IFN-γ production by CD8 + T cells and therefore compromised anti-viral immunity during influenza infection (Abt et al., 2012).

Recently, accumulating studies started to address the relationship between gut microbiota and lung disorders, which referred as the “gut-lung axis” (Marsland et al., 2015). It has been suggested that surviving bacteria, metabolites produced by gut bacteria or other factors may travel along the mesenteric lymphatic system to the circulatory system and subsequently enter pulmonary circulation, which may lead to the local activation of immune responses (Bradley et al., 2017). Numbers of examples reported alternations in the composition of the human microbiome during *M. tuberculosis* infection both in patients and in animal models (Winglee et al., 2014; Hu et al., 2019), and interaction between commensal microbiota and the host immune system to the pathogens. IFN-γ producing CD4 + T cells provide the major effector response to TB and IFN-γ is required for protection against disease progression in TB (Flynn et al., 1993). Based on the gut-lung axis theory, it is not surprising that there may be some stimuli that can originate in the gut, explaining the underlying mechanisms of resident microbiota regulating IFN-γ expression during TB infection.

MiRNAs are small non-coding RNAs that act as post-transcriptional regulators of mRNA by targeting specific RNAs for destruction or by repressing their translation (Kim et al., 2017). Recent researches had reported the regulation of cytokine production and function by miRNA (Ma et al., 2011). MicroRNA-21 (miR-21) has been identified as one of the most highly expressed miRNAs in various tumors (Pan et al., 2010) and has several reported functions that may impact the disease, including roles in both innate and adaptive immunity. In particular, miR-21 has been shown to modulate the responses of macrophages to bacterially derived Toll-like receptor (TLR) agonists including LPS. MiR-21 was also shown to be involved in inflammatory responses and regulate the immune responses by targeting programmed cell death 4 (PDCD4) (Frankel et al., 2008). Furthermore, Lu et al. (2011) also revealed that miR-21 can be induced in the lung of multiple asthma models and regulates lung eosinophilia, theTh1/Th2 balance and the prognosis for asthma. While the impact of bacterial pathogens in miRNA expression profile has been subjected to intensive investigation, our knowledge about the function of miRNAs in the GI tract is still very limited, especially about the mechanism by which and how miRNAs regulate gene expressions of inflammatory signaling such as IFN-γ.

An increasing number of studies indicate that miRNAs showed different expression patterns when the gut microbiota was altered and that differentiated miRNAs might contribute to shaping the interaction between the host and gut microbiota (Dalmasso et al., 2011a; Liu et al., 2016). In addition, germ-free (GF) and antibiotic-treated mice had significantly more fecal miRNA compared to controls associated with specific pathogen-free (SPF) microbiota, suggesting that gut microbes contribute to a specific miRNA signature. For example, Singh et al. (2012) identified 16 miRNAs that were differentially expressed in the cecum of GF and SPF mice. Their results further demonstrated that the targets of these miRNAs were genes that maintain the intestinal barrier. Another example is miR-107, previously reported to promote metastasis in colorectal cancer was differentially expressed in the cecum of GF mice compared to SPF mice (Xue et al., 2014). Therefore, these studies provide clues that the microbiota regulates host gene expression through modulation of the host miRNA signature.

More importantly, although altered miRNA expression profiles have been identified in serum and tissue from TB patients (Huang et al., 2015), the mechanistic basis underlying the development of TB by commensal bacteria through miRNAs has not been fully elucidated. Identifying the miRNAs regulated by gut microbiota of the production and function of IFN-γ will contribute to a better understanding of how to protect the host during *M. tuberculosis* infection while limiting the tissue damage caused by unrestrained inflammatory responses.

In the present study, we employed the “loss-of-function” model via the destruction/aberration of gut microbiota by antibiotic feedings and observed a similar broad-spectrum antibiotic regimen significantly decreases the gut microbiota and compromises immunity to pulmonary *M. tuberculosis* infection. Moreover, miR-21 was differentially expressed in the cecum and lung tissues while suppressing gut microbiome with broad-spectrum antibiotics during *M. tuberculosis* infection. Systemic antagonism of miR-21 provides effective protection against *M. tuberculosis* infection. On the basis of the observations noted above and the predication of IFN-γ as a potential target of miR-21, we demonstrated that miR-21 whose expression is remotely controlled by the microbiome directly targets IFN-γ and might in turn inhibit IFN-γ production leading to impaired anti-TB immunity. Thus, this work identifies a novel role for miR-21 in shaping the gut microbiota and the subsequent development of TB and may provide miR-21 as a novel target for the prevention and treatment of TB.

## Materials and Methods

### Key Resources Table

**Table:** 

REAGENT or RESOURCE	SOURCE	IDENTIFIER
Anti-Mouse CD3 APC-Cy7	Biolegend	Cat# 10033
Anti-Mouse CD4 FITC	Biolegend	Cat# 100406
Anti-Mouse CD8 Brilliant Violet 421	Biolegend	Cat# 100738
Anti-Mouse IFNγ PE	Biolegend	Cat# 505808
Fixation/Permeabilization Solution	BD Biosciences	Cat# 51-2090KZ
BD Perm/Wash Buffer	BD Biosciences	Cat# 51-2091KZ
Mouse Th1/Th2/Th17 Cytokine Kit	BD Biosciences	Cat# 560485
Lipofectamine 3000	Invitrogen	Cat# L3000008
Dual-Luciferase Reporter Assay System	Promega	Cat# E1910
Quick-Amp Labeling Kit	Agilent	Cat# 5190-0447
Gene Expression Hybridization Kit	Agilent	Cat# 5188-5242
Trizol	Invitrogen	Cat# 15596026
Middlebrook 7H11	BD Biosciences	Cat# 283810
Middlebrook OADC	BD Biosciences	Cat# 212240
EZNA Tissue DNA Kit	Omega Bio-Tek	Cat# D3396

#### Study Subjects

Samples of active pulmonary TB patients (*N* = 31) before anti-TB treatment were recruited by the Guangdong Provincial Center for TB Control. The healthy controls (HCs) (*N* = 21) were collected by the same hospital clinics with annual routine health examination final reports indicating healthy statuses. Patient information is summarized in Table 1. Informed consent from all patients included in the study was obtained according to protocols approved by the Internal Review and the Ethics Boards of Zhongshan School of Medicine of Sun Yat-sen University (SYSU).

**TABLE 1:** Demographic and clinical characteristics of active TB patients (TB) and healthy controls (HC).

	Healthy controls (HC)	TB patients (TB)
Sample size	21	31
Female	9 (42.9%)	12 (38.7%)
Male	12 (57.1%)	19 (61.3%)
Age (mean ± SD)	26.83 ± 9.8	27.47 ± 12.5
**Smear grade**		
None	21	11 (35.5%)
1+	N/A	9 (29.0%)
2+	N/A	5 (16.1%)
3+	N/A	6 (19.4%)
**Chest X-ray**		
Nodules and fibrotic scars	N/A	30 (96.8%)
Infiltrates or consolidations	N/A	30 (96.8%)
Cavity	N/A	9 (29.0%)
Atelectasis	N/A	7 (22.6%)
Pleural effusion	N/A	2 (6.5%)
Bronchiectasis	N/A	7 (22.6%)

*Recruitment site: Guangdong Center for Tuberculosis Control.*

#### Mice

All procedures were carried out under approval by the SYSU Institutional Animal care and Use Committee. SPF wild type of C57BL/6 (3-4 week) were obtained from the SYSU animal center and kept in SPF conditions at the Biosafety Level-3 laboratory of Sun Yat-sen University.

#### Antibiotics Treatment

A mix of ampicillin (1 mg/ml), neomycin (1 mg/ml), metronidazole (1mg/ml) and vancomycin (0.5mg/ml) were added in sterile drinking water of mice. Solutions and bottles were changed 2-3 times a week. Antibiotics activity was confirmed by cultivating the fecal pellets resuspended in BHI + 15% glycerol at 0.1g/ml on blood agar plates for 48 h at 37°C with in aerobic or anaerobic conditions. Mice were fed daily with broad-spectrum antibiotics during *M. tuberculosis* infection.

#### Tissue Pre-treatment for Bacteria Quantification

Lung and cecum tissues derived from mice of different groups were freshly isolated and broken into smaller pieces with a mortar and pestle, and homogenized in PBS buffer. The homogenate was treated with collagenase D (Sigma-Aldrich) (2 mg/mL final concentration) at 37°C for 15 min, followed by 10 min at 2000 × *g* at room temperature (RT). The pellet was washed with PBS (Sigma) (2000 × *g* for 10 min) and was further used for genomic DNA extraction.

#### DNA Extraction

Genomic DNA was isolated from tissue samples using the EZNA Tissue DNA Kit (Omega Bio-Tek) following the manufacturer’s instructions. The purity, concentration of DNA was detected by using the NanoPhotometer spectrophotometer and Qubit2.0 Fluorometer.

#### 16S rDNA Gene Amplification and Target Bacteria Analysis Using Quantitative PCR (qPCR)

Targeted qPCR systems were applied using Sybr Green for the total bacteria and the bacteria of interest in tissue samples with primers amplifying the genes encoding 16S rRNA or specific bacterial groups. qPCR of major representative genera of gut and lung microbiota as described previously was performed to ensure broad bacterial coverage (Bassis et al., 2015). Primers for target bacteria were chosen from previously published literature (Table 2). The abundance of genus in both groups in this study was calculated as the quantitative abundance of bacterial ΔCT values and was normalized to 16S rRNA expression, the internal reference using threshold cycle (CT) method (Schmittgen and Livak, 2008).

**TABLE 2:** The current list of qPCR primer couples.

Target group		Primer sequence (5′–3′)	Ref.
*Total bacteria*	F	CGGTGAATACGTTCCCGG	(Furet et al., 2009)
	R	TACGGCTACCTTGTTACGACTT	
*Actinobacteria*	F	TACGGCCGCAAGGCTA	(Trompette et al., 2014)
	R	TCRTCCCCACCTTCCTCCG	
*Bacteroidetes*	F	AACGCTAGCTACAGGCTTAACA	(Dick and Field, 2004)
	R	ACGCTACTTGGCTGGTTCA	
*Gammaproteobacteria*	F	CMATGCCGCGTGTGTGAA	(Mühling et al., 2008)
	R	ACTCCCCAGGCGGTCDACTTA	
*Prevotella*	F	CCTWCGATGGATAGGGGTT	(Layton et al., 2006)
	R	CACGCTACTTGGCTGGTTCAG	
*Veillonella*	F	GRAGAGCGATGGAAGCTT	(Tana et al., 2010)
	R	CCGTGGCTTTCTATTCC	
*Streptococcus pneumonia*	F	ACGCAATCTAGCAGATGAAGCA	(Chien et al., 2013)
	R	TCGTGCGTTTTAATTCCAGCT	
*Haemophilus*	F	AGCGGCTTGTAGTTCCTCTAACA	(Fukumoto et al., 2015)
	R	CAACAGAGTATCCGCCAAAAGTT	
*Blautia*	F	GTGAAGGAAGAAGTATCTCGG	(Kurakawa et al., 2015)
	R	TTGGTAAGGTTCTTCGCGTT	
*Neisseria*	F	CTGTTGGGCARCWTGAYTGC	(Yan et al., 2015)
	R	GATCGGTTTTRTGAGATTGG	
*Fusobacterium*	F	AAGCGCGTCTAGGTGGTTATGT	(Dalwai et al., 2007)
	R	TGTAGTTCCGCTTACCTCTCCA	
*Bacteroides thetaiotaomicron*	F	GACCGCATGGTCTTGTTATT	(Haugland et al., 2010)
	R	CGTAGGAGTTTGGACCGTGT	
*Escherichia coli*	F	CATGCCGCGTGTATGAAGAA	(Huijsdens et al., 2002; Ramirez-Farias et al., 2009)
	R	CGGGTAACGTCAATGAGCAAA	
*Faecalibacterium prausnitzii*	F	GGAGGAAGAAGGTCTTCGG	(Ramirez-Farias et al., 2009)
	R	AATTCCGCCTACCTCTGCACT	

#### Fecal Microbiota Transplantation (FMT)

Fecal samples (200–300 mg) from 10 healthy individuals were collected separately, homogenized and resuspended with 15% glycerol/PBS followed by centrifuge s at 2000 rpm for 10 min at 4°C. The supernatant slurry was pooled together and was aliquoted and stored at -80°C for future use. SPF mice were firstly treated with antibiotics for 1 week and colonized with fecal microbiota 2 days later. the fecal materials were thrawed in PBS, and 0.2 ml of the suspension containing a fecal microbiota obtained from healthy individuals, was transferred by oral gavage with a gap of 3 days interval into each mouse to reconstitute the gut composition.

#### *M. tuberculosis* Infection and Bacterial Count

Six to eight-week-old C57BL/6 mice were challenged intraperitoneally (i.p) with frozen stocks of wild type *M. tuberculosis* (H37Rv) strains, using an infection dose of 2.5 × 10^5^ CFUs per mouse in 0.2 ml of suspension as described in our previous study (Wang et al., 2015). Lungs were carefully homogenized and the numbers of viable bacteria in the lungs were monitored by plating 10-fold serial dilution of lung homogenates from individual mice on to Middlebrook 7H11 (BD Biosciences) agar plate. The CFUs were counted after 21 days incubation at 37°C.

#### Histopathological Analyses of *M. tuberculosis*-Infected Mice

At the end of the *M. tuberculosis* infection period, animals were sacrificed. Lung tissues of mice were fixed in 10% formalin and processed for paraffin embedding. Paraffin Sections of 5 μm were counterstained with hematoxylin and eosin (H&E). Images were obtained using a microscope (BX51WI, Olympus) and a digital camera (DP30BW, Olympus). All sections were interpreted by the same person and scored semiquantitatively, blinded to the variables of the experiment. The histopathological parameters inflammatory lesions and granuloma formation were scored as absent (0-2), minimal (2-4), slight (4-6), moderate (6-8), marked, or strong (8-10) respectively. In this score, the frequency, as well as the severity of the lesions, were incorporated. Granuloma formation was scored by estimating the occupied area of the lung section. The lungs of three animals were examined and the mean score of each of the four histological parameters was calculated.

#### MicroRNA Sequencing, Data Analysis and Statistics

Total RNA of lung and cecum in *M. tuberculosis*-infected mice with or without the treatment of antibiotics were isolated with Trizol reagent (Invitrogen Life Technologies) and used for further miRNA microarray analysis. Briefly, we first labeled the RNAs with fluorescent probes by the Quick Amp Labeling Kit (Agilent), evaluated the purity and quality of the labeled RNA, and further hybridized using the Agilent Gene Expression Hybridization Kit (Agilent). The miRNA chips were washed with Gene Expression Wash Buffer (Agilent) and were then scanned with an Affymetrix GeneChip Scanner to determine expression profiles of miRNA in the lungs and cecum from treated or untreated mice. Those miRNAs with the differential fold changes of expression are larger as 2 as (or smaller than 0.5) and *P* < 0.05 using an independent *t*-test by R statistical software between the two groups were considered as significant and chosen for further bioinformatics analysis.

#### Design and Administration of miR21-Antagomir, Antagomir Control in Mice

miR21-agomir, antagomir and their control were designed by Ribobio corporation and purchased commercially. Before infection with *M. tuberculosis* (H37Rv), each mouse received i.p. injection of 15nmol of miRNA-21 agomir, antagomir and control for a total of four dosages with an interval of 3 days between two dosages.

#### Flu a Infection for miR-21 Quantification

Five to seven-week-old mice were anesthetized by intramuscular injection of 1.25 mg of ketamine plus 0.25 mg of xylazine in 100 μl of phosphate buffered saline (PBS), and then intranasally (i.n.) infected with 50 μl of PBS containing (or not, in a mock sample) 200 p.f.u. of the H1N1 IAV strain A/WSN/1933. Mice were fed daily with broad-spectrum antibiotics during influenza A infection. Animals that had lost >20% of their original body weight were euthanized. Total RNA was extracted from lungs of influenza A-infected or mock-infected mice and processed for qPCR quantification of miR-21.

#### Quantitation of miR-21 Expression

Total RNA was prepared from tissues or PBMCs using Trizol reagent. The concentration of total RNA was determined by a Nanodrop spectrometer. The cDNA was prepared by reverse transcription using a miR-21 specific primer or a U6 control primer, and real-time qPCR was performed as previously described (Wang et al., 2015). For SYBR-Green amplification, a melting step was added. Quantitative-PCR data were normalized to the expression levels of U6.

#### Luciferase Reporter Assay

The targets of miR-21-3p were identified by bioinformatic software Targetscan online. For dual-luciferase reporter assay, the IFN-G 3′ UTR segments, containing the binding elements of miR-21-3p or its mutant versions were synthesized commercially. Plasmid DNA with desired firefly luciferase reporter and pRL-TK renilla plasmids along with miRNA mimic or inhibitor were cotransfected into HEK293T cells using lipofectamine 3000 (Invitrogen, Carlsbad, CA, United States). Relative activity of firefly luciferase unit (RLU) at 48 h post-transfection using a dual-luciferase reporter assay kit as recommended by the manufacturer (Promega, Madison, WI, United States) as instructions. A Renilla luciferase-expressing plasmid pRL-TK (Promega, Madison, WI, United States) was included in the transfection to normalize the efficiency of each transfection.

#### Intracellular Flow Cytometric Detection of IFN-γ Production in CD4+ and CD8 + T lymphocytes in Lungs

Experiments were conducted as described in our previous publication (Wang et al., 2015). Briefly, lungs were isolated and cut into small pieces and digested with collagenase D at the time of killing (Sigma). Then lung tissues were washed with PBS, carefully homogenized and filtered over a 70-μm filter to obtain single-cell suspensions. Lung lymphocytes were resuspended in RPMI 1640-based media containing 10% fetal calf serum and seeded (10^6^cells per ml) into 24-well plates (Costar). Cells were firstly incubated in the presence of *Mtb* antigens (rESAT-6) for 16 h followed by another 6 h 3 μg/ml anti-CD28 MAb stimulation (Chackerian et al., 2001; Cardoso et al., 2002). After the incubation period, the cells were harvested, resuspended in PBS-fluorescence-activated cell-sorting (FACS) buffer and cell suspension was transferred into polystyrene round bottom tubes (BD) for surface staining. After staining for cell-surface markers for 20 min, cells were then permeabilized for 30 min (Cytofix/Cytoperm; BD) and stained 45 min for intracellular molecules such as IFN-γ before fixation with 2% formalin/PBS for 24 h. Cells were then analyzed using polychromatic flow cytometry. To ensure the specific immune staining of ICS, isotype IgG served as negative controls for staining surface markers or intracellular cytokines. Data were obtained from Beckman CytoFLEX S and analyzed by Kaluza 1.3 software. The gating strategy was as followed: the debris was first excluded by a morphology gate based on FSC-A and SSC-A. Then, non-singlets were eliminated from the analysis by a single cell gate based on FSC-H and FSC-A. Positive cells were gated with CD3 antigen and further divided based on their expression of CD4 and CD8 antigens.

### Cytometric Bead Array (CBA)

Previously described procedures were employed (Wang et al., 2015). Cells derived from mice with indicated treatments were seeded at a density of 1 × 10^6^ cells per well and stimulated with *Mtb* antigens (rESAT-6) at 37°C. Culture supernatants of cells were analyzed for the production of following cytokines: IL-2, IL-4, IL-6, IFN-γ, TNF-α, IL-17A, and IL-10 using the cytometric bead assay (CBA) mouse Th1/Th2/Th1 cytokine kit (BD) according to the manufacturer’s instructions. Data were acquired on the Beckman Coulter Gallios (Beckman). The concentrations of IFN-γ production were revealed by the fluorescence intensity and were calculated relative to the standard dilution curve.

### Statistical Analysis

All statistical tests were performed using Prism software. Unless otherwise indicated, the non-parametric Mann–Whitney *U*-test was often used for two groups comparisons to avoid the assumption of a normal distribution. For samples more than 20, statistical analyses were performed with the unpaired two-tailed Student *t-test* as indicated in the figures. The *P* values of significant differences are reported. Plotted data represent means ± SD unless otherwise stated. All experiments were replicated in the laboratory at least two times. In figure legend, N represents the number of samples. ^∗^*p* < 0.05, ^∗∗^*p* < 0.01, ^∗∗∗^*p* < 0.001, and ^****^ *p* < 0.0001. NS, no statistical significance.

## Results

### Dysbiosis of Gut Microbiota Provoked Susceptibility to TB Infection

Recently, human microbiota habitat in the body such as gut and respiratory tract, have been characterized by high-throughput sequencing technologies. Recent studies demonstrate that the lung is not sterile contrary to previous belief (Mathieu et al., 2018). Moreover, accumulating studies show that disturbance in gut microbiota is associated with the onset and progression of many diseases such as inflammatory bowel disease, autoimmunity, obesity and cancer (Sanapareddy et al., 2012; Vetizou et al., 2015; Gray et al., 2017) by calibrating the host immunity (Schirmer et al., 2016; Thiemann et al., 2017). Besides, in addition to its local effects, gut microbiota modulates host immune response at extra-intestinal sites such as the brain, bone marrow and lung (Round and Mazmanian, 2009; Abt et al., 2012; Hooper et al., 2012; Arpaia et al., 2013; Atarashi et al., 2013; Honda and Littman, 2016; Schirmer et al., 2016). A key parameter controlling susceptibility to TB is the balance between the virulence of the *M. tuberculosis* strain and the immune status of the infected host. We therefore hypothesized that microbiome dysbiosis would have a profound impact on the microbiota that alters the nutritional landscape of the gut or the lung and increase the host susceptibility to TB due to aberrant immune response (Modi et al., 2014; Zanvit et al., 2015).

Antimicrobials remain the major and most potent factor causing dysbiosis (Moos et al., 2016), which refers to the microbial imbalance or maladaptation. As an initial step to investigate the role of microbiota dysbiosis in TB infection, we initially employed the “the-loss-of-function” model by antibiotics feedings consisting in the administration of a cocktail of broad-spectrum antibiotics composed of ampicillin, neomycin sulfate, metronidazole, and vancomycin for a week and determined the abundance and compositions of the microbiota. These antibiotics exhibited no impact on *M.tuerbculsosis* growth in a Middlebrook 7H11 agar formulation (Chang et al., 2002). Surprisingly, the significant loss of abundance of microbiota was only observed in the gut but not in the lung of the antibiotics-fed mice when compared with that of water-fed mice (Supplementary Figure 1). Numerous studies have agreed that gut and lung microbiota were dominated by *Firmicutes, Bacteriodetes, Proteobacteria*, and *Actinobacteria phyla*. Moreover, it was reported that genus *Streptococcus, Haemophilus, Veilonella*, and *Prevotella* (Clemente et al., 2012; Lozupone et al., 2012; Mathieu et al., 2018) were the dominated genera in the lung while genus *Neisseria, Fusobacterium, Escherichia* and *Bacteroides* accounted for 50–100% of the gut microbiota (Maslowski and MacKay, 2011). Thus, to further assess the effect of antibiotics on gut and lung microbiota communities, we characterized the levels of the representative phyla and genera dominated in gut and lung bacterial communities by qPCR between the antibiotics-treated and untreated group to ensure coverage of bacteria composition. The levels of lung microbiota communities have been slightly altered while gut microbiota demonstrated a marked decrease in the majority of the bacteria at both phyla and genus levels (Supplementary Figure 2). There were no significant differences in the composition of lung microbiota with or without antibiotics treatment (Supplementary Figure 2A), which was consistent with one study reporting that the effect on the lung microbiome is relatively minor, insignificant, and temporary in response to *M. tuberculosis* infection (Namasivayam et al., 2017). Moreover, the relative abundance of the most abundant phyla *Bacteriodetes*, which accounts for over 40% of the total gut microbiota, was significantly decreased in antibiotics-fed mice. Likewise, a similar trend was observed for the other phyla or genus. Whereas *Actinobacteria* is the only phyla, with no significant alternations of proportions by qPCR in the overall population of gut microbiota (Supplementary Figure 2B). Thus, our qPCR analysis of microbiota communities in gut and lung confirmed that antibiotics treatment only altered gut microbiota but had little effect on lung microbiome. Hence, to study anti-TB response to microbiota dysbiosis, we would focus on the commensal bacterial in the gut instead of the lung microbiome.

**FIGURE 1:**
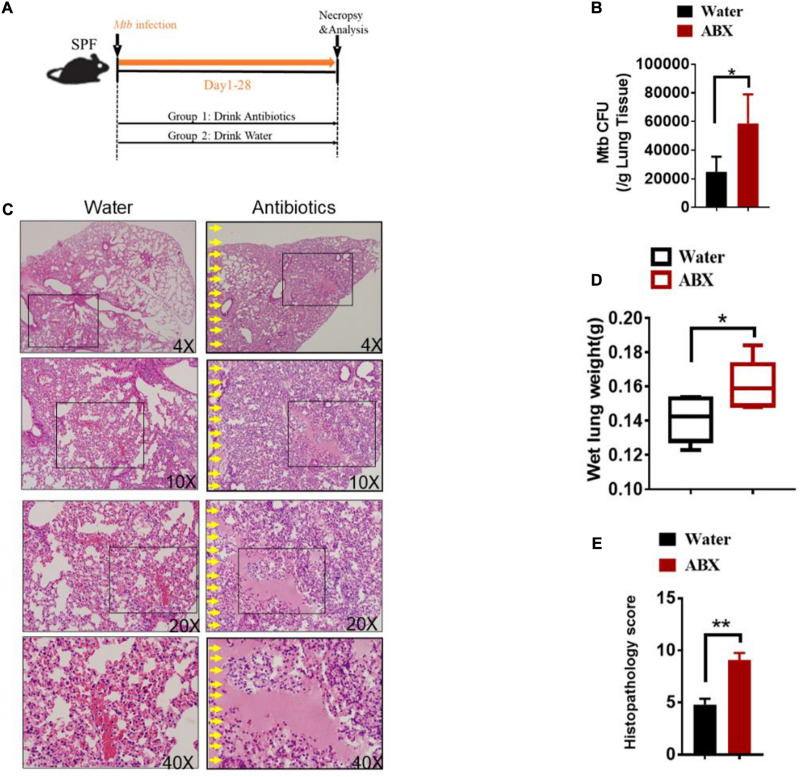
Alteration in the gut microbiota provokes susceptibility to Tuberculosis. **(A)** Experimental design to test the importance of gut microbiota in control of *M. tuberculosis* infection and TB pathology. *M. tuberculosis*-infected mice were treated with sterilized water only (*N* = 6) or with broad-spectrum antibiotics (*N* = 6) [ampicillin (1 mg/ml), neomycin (1 mg/ml), metronidazole (1 mg/ml) and vancomycin (0.5 mg/ml)] in their drinking water for 4 weeks until mice were sacrificed for immunological, pathological, and bacillus burden analyses. **(B)** *M. tuberculosis* CFU quantitative analyses in the lungs (per gram) and cecum (per gram) in *M. tuberculosis*-infected mice with or without drinking antibiotics. **(C)** Hematoxylin and eosin (H&E) staining of representative lungs of *M. tuberculosis*-infected mice with or without drinking broad-spectrum antibiotics. Yellow arrows mark extensive damages of pulmonary structures and unresolved hemorrhage, and extensive infiltration of inflammatory cells in the pulmonary compartments observed in the lung sections derived from *M. tuberculosis*-infected mice with drinking antibiotics. **(D)** The values of wet lung weight (g) for six representative lungs derived from *M. tuberculosis*-challenged mice with drinking antibiotics or water. **(E)** Quantitative representation of the lung histopathology in mice with antibiotics treatment or water treatment after infection with *M. tuberculosis* analyzed after hematoxylin and eosin (H&E) staining using histopathology score scale shown in Supplementary Figure 4. The histopathology score was averaged for six mice per each experimental group per time point. Data shown as mean ± SEM are representative of two independent experiments (*N* = 6 animals/group), *p < 0.05, **p < 0.01, non-parametric Mann–Whitney *U*-test.

To evaluate the role of antibiotics on the development of TB infection, we further investigated whether antibiotics would directly affect lung pathology by hematoxylin and eosin (H&E) staining of lung sections without *M.tuberculosis* infection. Indeed, no obvious differences were observed in the lungs derived from antibiotics-treated and untreated mice (Supplementary Figure 3). We then determined whether gut microbial dysbiosis by daily broad-spectrum antibiotics feedings would compromise the ability of gut microbiota to support or sustain immune response or immunity against *M. tuberculosis* infection (Figure 1A). When compared with water controls, antibiotics feedings increased *M. tuberculosis* burdens in the lungs (Figure 1B), which was further corroborated with the histopathological analysis of the lungs (Supplementary Figure 4). More importantly, antibiotics treatment led to more severe lung pathology characterized by more cystic changes, hemorrhage, necrosis observed in the lungs (Figures 1C–E).

Thus, these data collectively suggested that the host gut microbiota but not lung microbiota contributes to resistance to TB and is critical in controlling *M. tuberculosis* infection and pathology.

### Gut Dysbiosis Significantly Up-Regulated the Expression of miR-21

**FIGURE 2:**
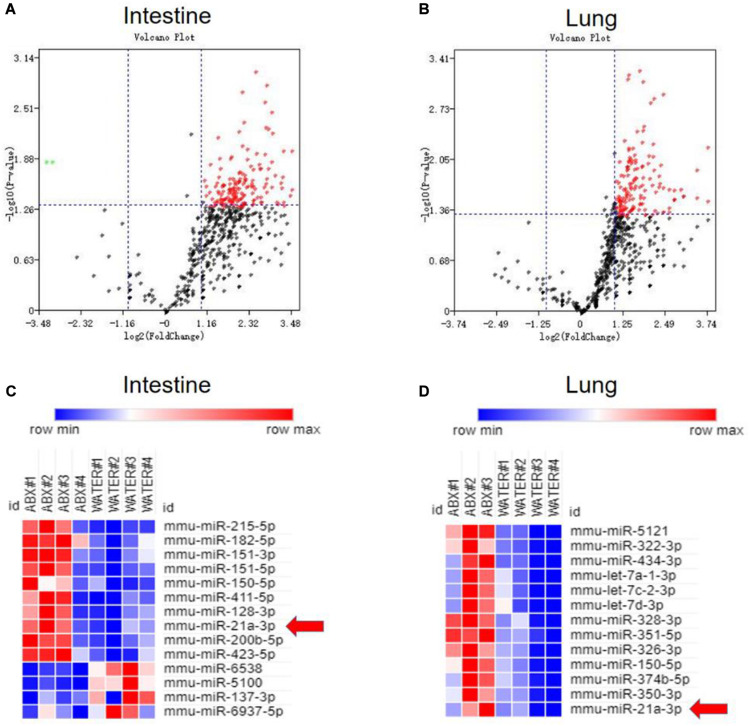
Commensal bacteria regulate miRNA expression in cecum and lungs. Total RNA, including low molecular weight RNA, was prepared from the cecum and lungs derived from mice with or without antibiotics treatment. The expression of miRNA was analyzed by *t*-test. Volcano Plots compare the expression of miRNAs between cecum in mice with or without antibiotics treatment **(A)** or between lungs in mice with or without antibiotics treatment **(B)**. The most significantly differentially expressed miRNAs with a greater than 2-fold difference in expression in cecum and lungs are shown in panels **(C,D)**.

Our and others’ previous studies on lncRNA (Wang et al., 2015) and miRNA (Ma et al., 2011) suggested that non-coding RNAs can modulate both innate and adaptive immunity in response to *M. tuberculosis* infection. Moreover, an increasing number of pieces of evidence indicated that miRNAs showed different expression patterns when the gut microbiota was altered and that differentiated miRNAs might contribute to shaping the interaction between the host and gut microbiota. Therefore, we would ask whether gut bacteria were associated with active TB infection by mediating the expression of some miRNAs. To clarify the postulation, we analyzed the miRNA expression profile in lungs and cecum of antibiotics treated and untreated mice in response to *M. tuberculosis* infection. We found differentially expressed miRNAs both in the lungs and cecum in the intestinal dysbiosis group. Of the 1767 miRNAs assayed, 150 were found to be significantly up-regulated, in the lungs of antibiotics-treated mice compared with control mice. Moreover, 190 out of 1019 miRNAs were found to be significantly up-regulated while 2 were down-regulated in the cecum of antibiotics-treated mice. Volcano plot analysis showed that miRNA differentially expressed in the lungs and cecum between microbiota dysbiosis mice and healthy mice with the *p-*values < 0.05 and fold of change >2 (Figures 2A,B). Notably, miR-21-3p was identified as one of the most significantly differently expressed miRNAs in both lungs and cecum in the microbiota dysbiosis mice (Figures 2C,D).

The remarkable up-regulation of the miR-21-3p observed in the lung and the cecum during TB infection/disease raises a possibility to examine whether this induction is somehow linked to alternations of gut microbiota by antibiotics feeding or due to antibiotics themselves. To address this question, we then assessed miR-21-3p expression by qPCR using an *in vitro* HEK-293T cell system. Not surprisingly, no significant difference was observed in miR-21-3p expression in HEK 293T cells with or without antibiotics treatment (Supplementary Figure 5). Thus, antibiotics themselves would not influence the expression pattern of miR-21-3p.

Our results collectively suggested that gut microbiota indeed acted as a regulator of miR-21-5p and up-regulated miR-21-3p in lungs and cecum is one of the key immunological markers in TB infection/disease associated with gut dysbiosis, and such up-regulation of miR-21-3p might indeed somehow be involved in regulating TB pathogenesis.

### MiR-21-3p Expression Was Induced During Active TB Infection and Dictated by Commensal Bacteria

**FIGURE 3:**
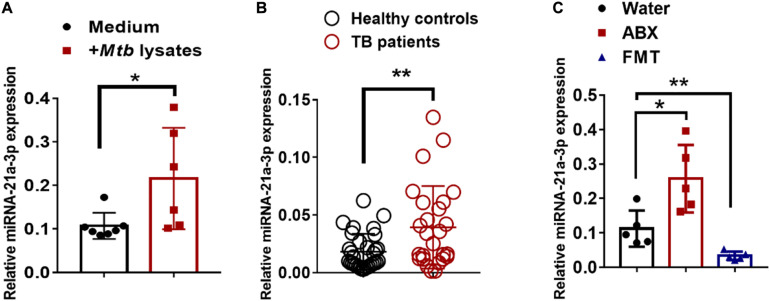
MiR-21-3p expression deregulated by bacterial components is induced by *M. tuberculosis* infection in humans and mice. **(A)** MiR-21-3p expression in PBMCs from healthy controls (*N* = 21) and active TB patients (*N* = 31) was analyzed by qPCR. **(B)** Splenocytes from mice were stimulated with Medium (*N* = 7) or *M. tuberculosis* antigen (*N* = 6) for 72 h. MiR-21-3p expression was analyzed by qPCR. Normalized values of miR-21-3p with or without stimulation are shown as the mean ± SD of 3 independent experiments. **(C)** MiR-21-3p expression of representative lungs derived from the *M. tuberculosis*-infected mice with treatment of water (*N* = 5), antibiotics (*N* = 5) or FMTs of gut microbiota from healthy individuals (*N* = 5), respectively. Data shown as mean ± SEM are representative of two independent experiments. For human study, *N* > 20/per group, **p < 0.01, Student *t*-test. For animal experiments, *N* = 5-7 mice/per group *p < 0.05, non-parametric Mann–Whitney *U*-test.

We then questioned whether gut dysbiosis-induced miR-21-3p was also associated with TB infection/disease in humans, we first investigated the expression of miR-21-3p in the context of *M. tuberculosis* infection in humans by qPCR. Indeed, high consistency of qPCR validation of miR-21-3p in cohorts of TB patients and healthy controls further supported that up-regulation of miR-21-3p might somehow be linked to active TB disease (Figure 3A). Such induction of miR-21-3p expression was at least partially specific for *M. tuberculosis* (Figure 3B) as no difference of miR-21-3p expression was observed in response to flu A infection (Supplementary Figure 6).

Recent evidence has reported changes in the gut microbial composition on *M. tuberculosis* exposure (Hu et al., 2019). Since it had been suggested that gnotobiotic mice might have specific expression patterns of miRNA profile, it was likely that miR-21-3p expression might constitute a signature that reflects the statuses in the gut bacteria ecosystem. We postulated that the “the-gain-of-function” model via oral transfer in mice would help to define the associations between gut dysbiosis and extra-intestinal miR-21-3p expression. We reconstituted the gut communities by oral gavage of human microbiota to mice. Interestingly, we observed a significant decline in the number of gut microbes in antibiotics-treated mice whereas the abundance of gut microbes was restored about 80% when compared with that of normal mice after fecal transplantation (Supplementary Figure 2B). It suggested that lowering microbial diversity by antibiotics treatment could not be fully restored to its original frequency by fecal transplantation for a short time. We also observed a significant increase in the number of *Bacteroidetes* and *Veillonella* phyla, but a slight decline in the level of *Actinobacteria* phyla (Supplementary Figure 2B). These results further support the partial reconstitution of gut microbial composition through fecal materials transfer. More importantly, alternations of the proportions of gut communities were seen, indicating the different diversity of gut microbes between humans and mice.

We further examined whether the reconstitution of gut microbiota from healthy individuals to mice would result in changes in miR-21-3p expression. Groups of mice were fed three times via oral gavage with water or fecal materials from healthy controls, the expression of miR-21-3p in the lungs was evaluated for changes by qPCR. Indeed, FMT was correlated with a decrease in the expression of miR-21-3p, which can be interpreted as the effect of gut microbiota on remotely regulating miR expression profiles (Figure 3C). Thus, our result further suggested that the gut microbiota plays a crucial role in regulating the expression of miR-21-3p in the lungs.

### MiR-21 Played Critical Roles During *M. tuberculosis* Infection

**FIGURE 4:**
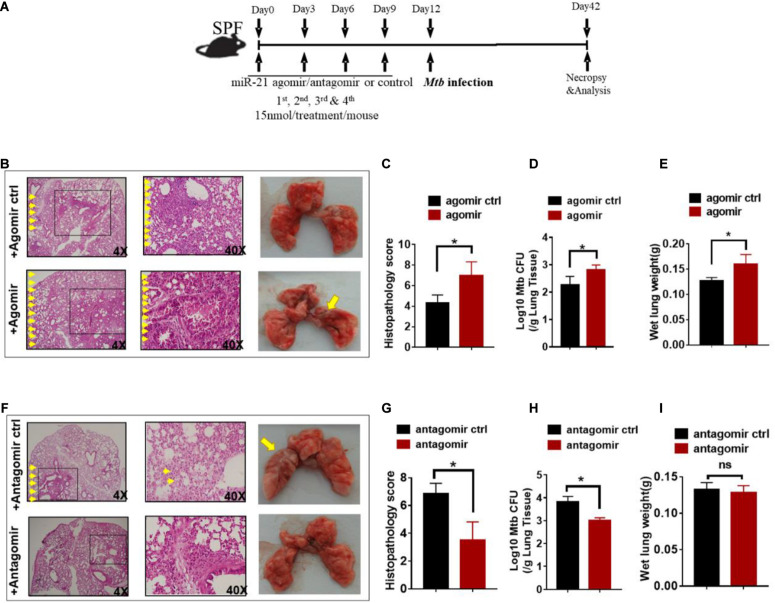
Exogenous miR-21-3p contributes to more apparent *M. tuberculosis* infection while antagonism of miR-21-3p enhances immune protection against *M. tuberculosis* infection. **(A)** Experimental diagram showed that *M. tuberculosis*-infected mice were treated with agomir, antagomir or their controls in the presence of antibiotics and sacrificed at the indicated time point. Mice were i.p.- injected 4 doses of miRNA-21 agomir or agomir control with an interval of 3 days followed by *M.tuberculosis* infection. **(B–E)** Determination of the effect of exogenous miRNA-21 on *M. tuberculosis* infection. **(B)** Hematoxylin and eosin (H&E) staining and gross pathology of representative lungs of *M. tuberculosis*-infected miRNA-21 agomir treated (*N* = 3) or agomir control-treated (*N* = 3) mice; **(C)** Quantitative representation of the lung histopathology in mice with exogenous agomir or control treatment with *M. tuberculosis* infection analyzed after hematoxylin and eosin (H&E) staining using histopathology score scale is shown. **(D)** CFU of *M. tuberculosis* was analyzed in the lungs (per gram) treated with miRNA-21 agomir or miRNA-21 agomir control in infected mice. **(E)** The values of wet lung weight (g) for representative lungs derived from *M. tuberculosis*-challenged mice with agomir or agomir control. **(F–I)** Determination of the effect of antagonism of miRNA-21 on *M. tuberculosis* infection. Mice were treated in the same way as antagomir or antagomir control before *M.tuberculosis* infection. **(F)** H&E staining and gross pathology; **(G)** Lung pathology score; **(H)** CFU analysis; **(I)** wet lung weight (g) in representative lungs derived from mice with antagomir or antagomir control treatment. Data shown as mean ± SEM are representative of two independent experiments (*N* = 3 animals/group), *p < 0.05, ns: no statistical significance, non-parametric Mann–Whitney *U*-test.

Since gut microbiota was critical for developing resistance to TB and increased miR-21-3p expression patterns correlated with active TB infection in humans, we hypothesized that miR-21-3p, regulated by commensal gut bacteria might promote or depress the ability of gut microbiota to regulate pulmonary response and immunity during *M. tuberculosis* infection. We then investigated whether *in vivo* intervention of miR-21-3p expression has an impact on *M. tuberculosis* infection in mice. Before infection with *M. tuberculosis*, mice were firstly treated with either miR-21-3p agomir, antagomir or their controls by i.p. injection, then followed by a total of three dosages with an interval of 3 days between two dosages (Figure 4A). Exogenous expression of miR-21-3p by inoculating miR-21-3p agomir results in increased *M. tuberculosis* burdens in lungs and induced more severe TB pathology (Figures 4B–E). Meanwhile, we assessed whether miR-21-3p is required for restricting *M. tuberculosis* replication inhibition. Remarkably, mice that received miR-21-3p antagomir displayed significantly attenuated tissue pathology and significantly decreased *M. tuberculosis* burdens, while control-treated mice showed more severe lung pathology characterized by large-scale damage of lung structure (Figures 4F–I). Thus, these results illustrated that miR-21-3p plays a critical role in *M. tuberculosis* infection and TB pathology, and gut bacteria may mediate host resistance against TB through inhibition of miR-21-3p expression in lungs.

### MiR-21 Impaired Protective Immunity by Affecting IFN-γ Production During *M. tuberculosis* Infection

**FIGURE 5:**
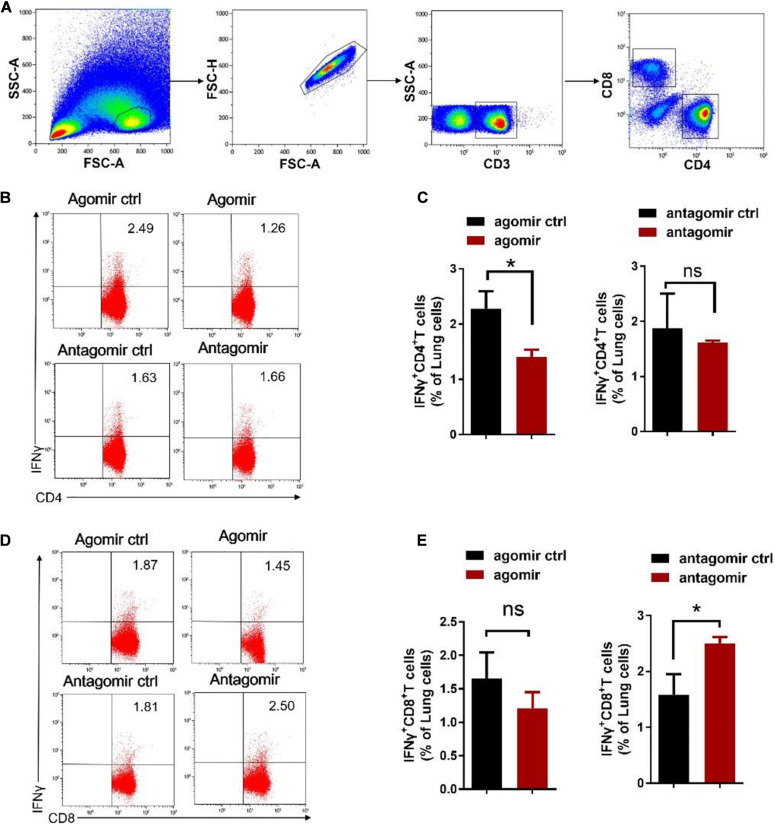
Exogenous miR-21-3p inhibits CD4 + IFN-γ + subsets while antagonism of miR-21-3p eliciting CD8 + IFN-γ + in the lungs of *M. tuberculosis*-infected mice. **(A)** Gating strategy used for the analysis of IFN-γ expression on CD4 + and CD8 + T cells. Dot-plots show lung lymphocytes gating on the forward (FSC) and side scatter (SSC) detectors. Live CD3 + T-cells were gated from lymphocytes after single cells gating, followed by identification of CD4 + and CD8 + T-cells sub-populations. Arrows show the sequence of the gating used, starting from the lymphocytes gate. **(B–E)** Representative flow cytometric dot plots and pooled bar graphic data show the expression of IFN-γ of panels **(B–C)** CD4 + and **(D–E)** CD8 + in lung derived from *M. tuberculosis*-infected mice with agomir, antagomir and their corresponding controls. Lung lymphocytes were stimulated *in vitro* with *Mtb* antigen ESAT-6 (5 μg/ml) and anti-CD28 antibody. After the incubation period, cells were harvested and evaluated by flow cytometry. Cells were acquired in the gated lymphocyte population, and the quadrants were set up with an isotype-matched control. The numbers in the upper right quadrant of each dot plot are the percentages of CD4+ or CD8 + T cells produced IFN-γ. Data shown as mean ± SEM are representative of two independent experiments (*N* = 3 animals/group), *p < 0.05, ns, no statistical significance, non-parametric Mann–Whitney *U*-test.

We then examined how commensal bacteria-regulated miR-21 was involved in mediating protective immunity against *M. tuberculosis* infection. It is noteworthy that IFN-γ is required for immune resistance to TB (Flynn et al., 1993), and gut microbiota is important for the production of IFN-γ (Schirmer et al., 2016). We therefore postulated that IFN-γ might be a key mediator linking gut microbiota/miR-21 and pulmonary immune response against TB. On day 42 after infection with *M. tuberculosis*, intracellular cytokine staining of lungs derived from mice received exogenous miR-21 or antagonist of miR-21 were performed to investigate the physiological importance of miR-21 in the regulation of IFN-γ-production. In order to enhance the sensitivity of cytokine detection, cells were stimulated *in vitro* with the mycobacterial antigens and anti-CD28 MAb to increase cytokine production by T cells, and the cells were processed for flow cytometry as described in Materials and Methods. CD3 + CD4 + cells and CD3 + CD8 + cells were first gated (Figure 5A), and the results indicated that a significant decrease in the number of IFN-γ-producing CD4 + cells occurred in mice treated with exogenous miR-21 upon stimulation (Figures 5B,C), in contrast, more IFN-γ-producing CD8 + cells were observed in mice inoculating with miR-21 antagomir than did those from the control mice (Figures 5D–E). We further confirmed the presence of intracellular cytokine IFN-γ using a cytometric bead array (CBA). Cells derived from mice with agomir or antagomir and their respective controls were stimulated with *Mtb* antigen ESAT-6 and incubated for 72 h. The levels of the cytokines IFN-γ, IL-10, IL-4 and IL-17A were analyzed in the cell culture supernatants by CBA. The results indicated that the level of IFN-γ was significantly reduced in the mice received miR-21-3p agomir when compared to the control (835.56 ± 53.52 vs. 23.14 ± 61.3, pg/ml, *P* < 0.05) (Supplementary Figure 8A), while more IFN-γ production was observed in mice inoculating with miR-21 antagomir than did those from the control mice (*P* < 0.05) (Supplementary Figure 8B). Therefore, these data suggest that miR-21 negatively regulates anti-mycobacterial immune responses.

### Direct Targeting of the 3′-UTR of IFN-γ mRNA by miR-21

**FIGURE 6:**
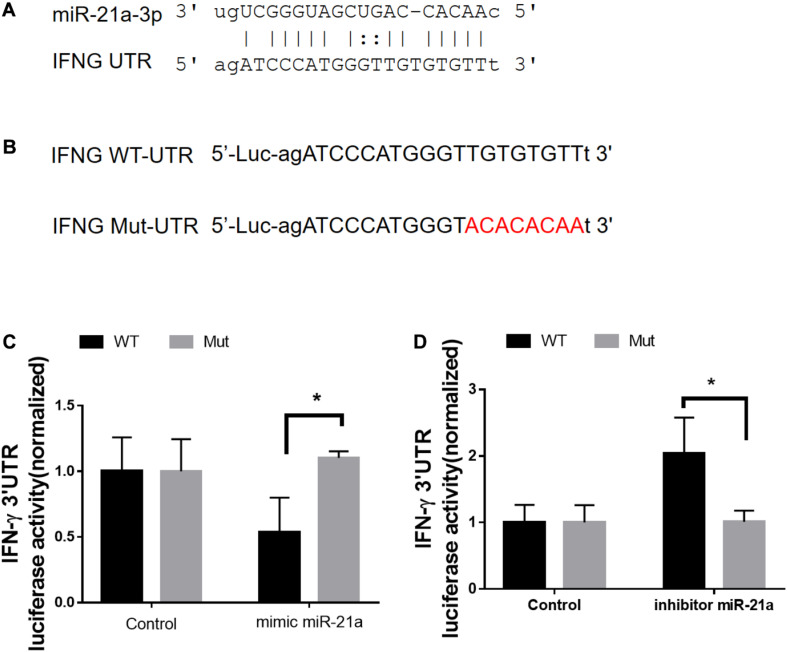
MiR-21a-3p directly targets IFN-γ mRNA 3′-UTR to inhibit its expression. **(A)** Predicted miR-21a-3p targeting sequence on the 3′-UTR of IFN-γ mRNA. **(B)** Wild type (IFN-γ-UTR) targeting sequence of miR-21a-3p on 3′-UTR of IFN-γ mRNA, and the mutated version (IFN-γ-mut), were cloned at the downstream of the luciferase open reading frame (Luc). **(C)** Luciferase activities of IFN-γ-UTR or IFN-γ-mut-UTR constructs were examined after the co-transfection of miR-NC or mimic miR-21a-3p, respectively. **(D)** Luciferase activities of IFN-γ-UTR or IFN-γ-mut-UTR constructs were examined after co-transfection of miR-NC or miR-21a-3p inhibitor, respectively. Data shown as mean ± SEM are representative of two independent experiments, *p < *0.05*, non-parametric Mann–Whitney *U*-test.

**FIGURE 7:**
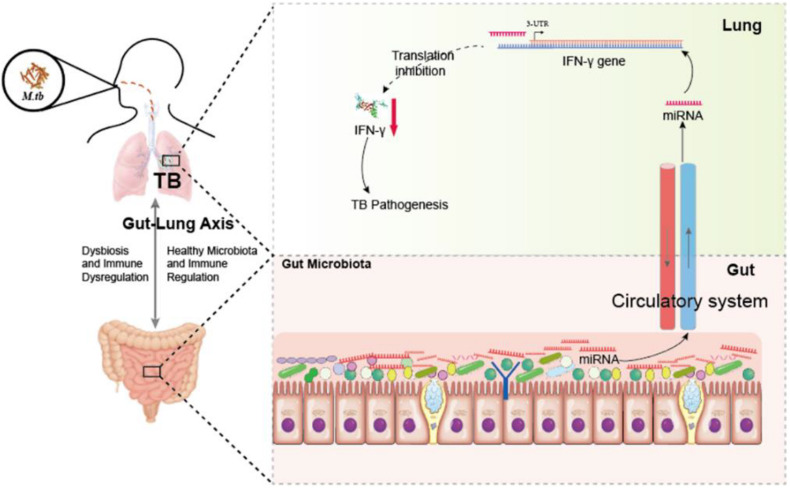
Hypothetic working model of regulating anti-TB protective immune paradigm via manipulating commensal bacteria-governed miRNA expression. Aberration of gut microbiota provokes susceptibility to TB and increases miR-21-3p expression not only in the cecum but also in the lungs during *M. tuberculosis* infection, in turn targeting IFN-γ. Exogenous miR-21-3p directly targets IFN- mRNA, in turn contributes to TB pathogenesis.

We next sought to investigate the molecular mechanisms by which antibiotics destruction of microbiota increased miR-21 expression and depressed IFN-γ responses leading to a loss of immune resistance to *M. tuberculosis* infection. Our above data suggested the potential inhibitory effect of miR-21-3p on IFN-γ production. We then hypothesized that miR-21 might directly target IFNG. We predicted the binding motif of miR-21a-3p on 3′UTR of IFNG by Targetscan database and revealed that 3′UTR of IFNG bears the miR-21 binding sites (Figure 6A). We then performed the luciferase reporter assay to verify the database predictions using plasmids containing a wild type as well as the mutated 3′UTR of IFNG-binding motif fused luciferase reporter (Figure 6B) and miR-21 mimic and inhibitor. Our results indicated that the luciferase activity of the IFN-γ 3′ UTR wild type reporter but not IFN-γ 3′ UTR reporter with mutated miR-21-binding sites was significantly inhibited when transfected the “mimics”of miR-21 (Figure 6C). In contrast, the endogenous miR-21 inhibitor significantly enhanced the luciferase activity of the IFN-γ 3′ UTR reporter, while no differences were found between cells transfected with the IFN-γ 3′ UTR reporter with mutated miR-21-binding sites with or without miR-21 inhibitor (Figure 6D). Thus, our data clearly illustrates that IFN-γ mRNA was targeted by miR-21a-3p, which is likely involved in the molecular mechanism underlying commensal bacteria-regulated miR-21 impaired anti-TB immunity (Figure 7).

## Discussion

Tuberculosis is the leading cause of death worldwide, killing nearly 2 million people each year (Maartens and Wilkinson, 2007). Discoveries of new paradigms for anti-TB immunity can certainly help develop new therapeutics and vaccines for global TB control (Zumla et al., 2013; Nunes-Alves, 2014; Rubin, 2014). Aerosol absorption of *M.tuberculsosis* is the natural route of TB infection by lung tissues. However, several studies affirmed that i.p. infection also results in chronic TB infection in mouse lungs similar to that observed during low dose aerosol infection with several advantages. The i.p. infection model establishes a steady-state level of CFUs in organs that do not change significantly over time (Orme and Collins, 1994; Phyu et al., 1998; Dahl et al., 2003). Given that clinical latency in humans is characterized by low bacillary loads, the i.p. route of infection may in fact model TB latency. Moreover, i.p. infections are low-level cross contamination between animals and the high-dose challenge with *Mycobacteria* is known to push the systemic immune responses. Therefore, the high-dose i.p. infection TB model may more appropriate for studying the gut microbiota shaped systemic immunity.

The microbiota plays an important role in the maintenance of intestinal homeostasis and a complex, reciprocal relationship with the host immune system (Lee et al., 2014) and so far, accumulating evidence had been highlighted the role of the microbial dysbiosis in contributing to the development and progress of various diseases and focused on identifying the underlying factors and mechanisms (Marchesi et al., 2016; Schirmer et al., 2016). Antibiotics are often used in clinics to treat bacterial infections, but they are also a major factor in disturbing the gut microbial composition and provoke host susceptibility to enteric infection (Modi et al., 2014). In the current study, we chose antibiotics, which are effective against both Gram-positive and -negative bacteria but not *M.tuberculosis* viability, to significantly induce dysbiosis in the gut (Chang et al., 2002). Interestingly, disruption of gut microbiota with antibiotics revealed a significant reduction of abundance and diversity of gut microbiota but not lung microbiota, indicating that antibiotics only influence the colonized bacteria at local organs. Moreover, key findings that emerged from the current study also revealed the role of gut microbiota in controlling the pathogenesis of TB as higher *M.tuberculosis* burden in the lungs and more severe pathology were observed with the treatment of antibiotics during TB. All histopathological parameters increased in severity in the course of TB infection. Thus, the severity of TB disease was evaluated by histopathological score. The histopathological scores were generally positively correlated with CFU counts and TB severity. Large differences in the antibiotics-induced lung pathology and more severe TB were observed, demonstrating further that intestinal dysbiosis might be a necessary factor contributing to the development and progress of active TB infection. This hypothesis gains support from our study and other reports wherein broad-spectrum antibiotics-driven microbial dysbiosis provokes susceptibility to TB in the mouse model (Khan et al., 2016).

Our results suggest a link between miRNA expression and gut microbes as we presented that intestinal dysbiosis by broad-spectrum antibiotics would significantly modulate the expression of miR-21-3p in lung and cecum derived from *M. tuberculosis*-infected mice. Previous investigations have also suggested the miRNA expression and gut microbes as the colonized gut microbiota in ileum and colon in SPF mice and Germ-free mice were different and such miRNAs would contribute to the development of diseases associated with dysbiosis of intestinal microbiota (Dalmasso et al., 2011b). More importantly, *Mycobacterium leprae* upregulates miR-21 expression to escape the vitamin D-dependent induction of antimicrobial peptides (Sheedy et al., 2010). Therefore, it is likely that altered miR-21-3p expression may partially result from host-microbe interactions and therefore involved in immune resistance against *M. tuberculosis* infection.

Somehow surprisingly, we found the gut microbiota can also impact host miRNA expression in the lungs well beyond the gut. The impact of antibiotics driven changes in gut microbiota and altered miR-21-3p expression was further supported by FMT reconstitution. Interestingly, fecal materials transplantation of antibiotics-treated mice restores the gut microbiota and increase miR-21-3p expression in their lungs. Consistently, two recent studies have also identified differential miRNA expression in the aorta and hippocampus of germ-free versus conventional mice. Results from us and others indicated that the commensal gut bacteria-related miRNA is linked to the regulation of the gut-lung axis for immunity and opened a novel and interesting avenue for future research.

It is noteworthy that miRNAs act as key regulators of innate and adaptive immunity as well as in disease development. More importantly, miRNAs recently reported to be governed by commensal bacteria, affected NF-kB-pathway activity via targeting the IL-23p19 gene (Xue et al., 2014). Notably, IFN-γ is required for immune resistance to TB (Flynn et al., 1993) and our results revealed regulation events from modulation of miR-21 in considerable IFN-γ responses that broadly involve CD4^+^ T and CD8^+^ T effector subpopulation. Thus, it is not surprising to see changes in IFN-γ- production and miR-21 mediated anti-TB immunity in our functional models. At the molecular level, the elevated IFN-γ production correlates with the upregulated expression of miR-21-3p. This negative correlation between miR-21-3p and IFN-γ is subsequently found to be a consequence of their directly targeting, where miR-21a-3p directly targets IFN-γ 3′-UTR to inhibit its expression.

In summary, our studies show an association between gut microbiota and *M. tuberculosis* infection and demonstrate a significant role for inhibition of miR-21-3p on controlling *M. tuberculosis* infection via modulating the immune protection via increasing production of IFN-γ. The current study defines regulatory pathways implicating intestinal dysbacteriosis induced-susceptibility to TB: intestinal dysbiosis→lung miRNA→targeting IFN-γ→impaired anti-TB immunity. Our work also presents a novel way toward new therapeutic intervention on controlling *M. tuberculosis* infection.

## Data Availability Statement

The datasets generated for this study can be found in the GSE125870.

## Ethics Statement

The animal study was reviewed and approved by the SYSU Institutional Animal care and Use committee.

## Author Contributions

FY and GZe led the project, conceived, and planned the experiments. YY and YC carried out the experiments. GL, GZh, LC, ZZ, QM, and GZe contributed to interpretation of the results. FY took the lead in writing the manuscript. All authors contributed to drafting the work and gave final approval for the version to be published, agreed to be accountable for all aspects of the work in ensuring that questions related to the accuracy or integrity of any part of the work are appropriately investigated and resolved and provided critical feedback and helped shape the research, analysis, and manuscript.

## Conflict of Interest

The authors declare that the research was conducted in the absence of any commercial or financial relationships that could be construed as a potential conflict of interest.
