# Supplementary material for: MiR-21 Is Remotely Governed by the Commensal Bacteria and Impairs Anti-TB Immunity by Down-Regulating IFN-γ
Source: Front Microbiol. 2021 Jan 21;11:512581. doi: 10.3389/fmicb.2020.512581 (PMC7859650; doi:10.3389/fmicb.2020.512581)
Supplement: Supplementary file 1 [file Data_Sheet_1.PDF]

# Figure S1

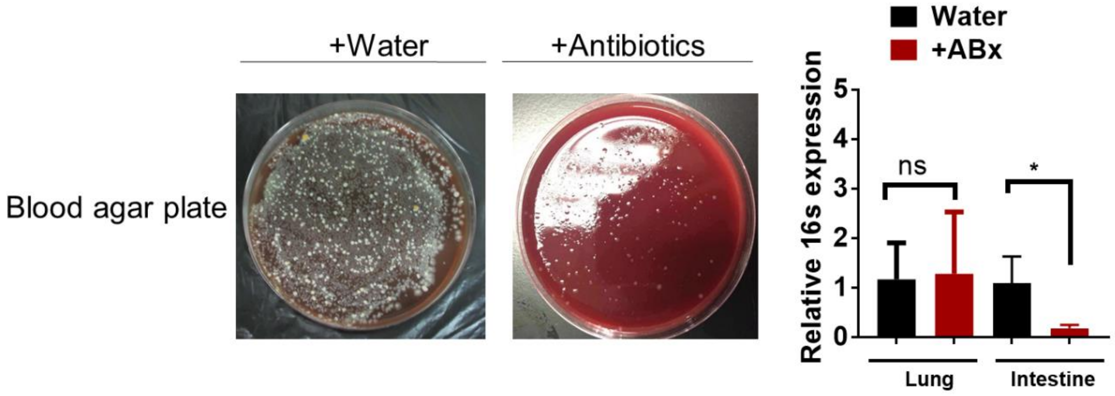


**Figure S1. Oral administrations of broad-spectrum antibiotics in *M. tuberculosis*-infected mice effectively reduce the abundance of gut microbiota but not lung microbiota.**

Fecal pellets derived from *M. tuberculosis*-infected mice with drinking antibiotics or water only were re-suspended in BHI+15% glycerol at 0.1g/ml and cultured on blood agar plates for 48 hours at 37°C. The 16s copies of total bacteria of lungs and gut derived from water-fed or antibiotics-fed mice were quantified using the qPCR. Error bars indicated average values± SEM. *$p$<0.05.

**Figure S2**

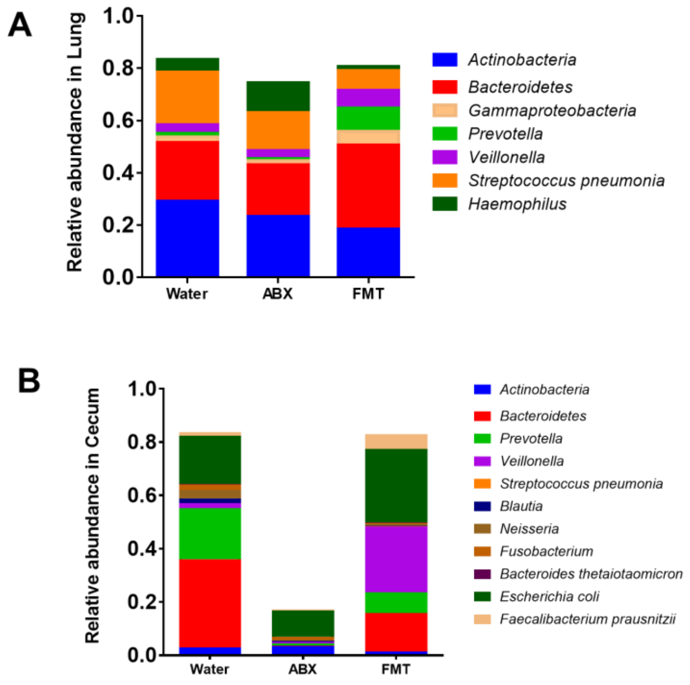


**Figure S2. Community structures of microbiota phyla and genus-level taxa in water, antibiotics-treated and FMT-treated mice.** Compositions of microbiota in the lung (A) and gut (B). Broad-spectrum antibiotics effectively reduce the compositions of gut microbiota but not lung microbiota whereas FMT to antibiotics-treated mice restores the gut community.

# Figure S3

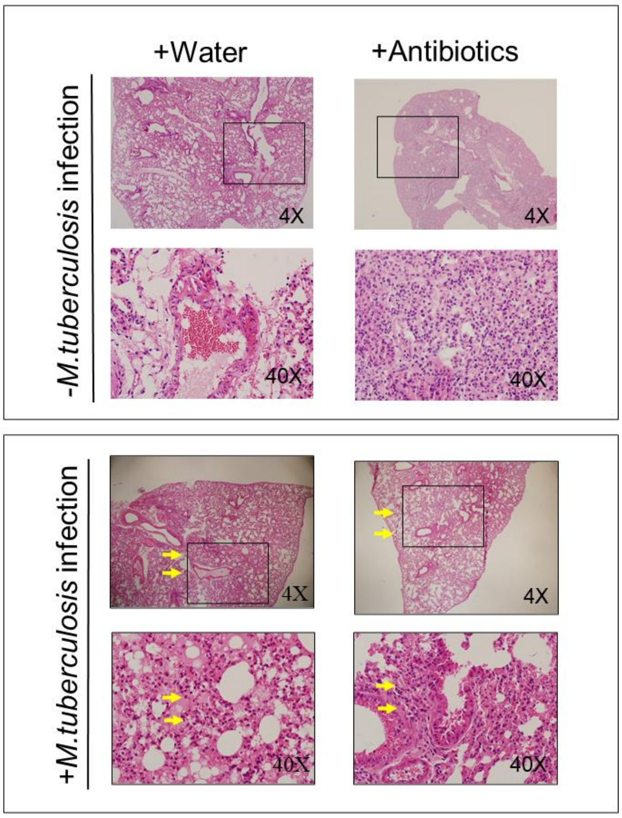


**Figure S3. Antibiotics treatment would not induce lung pathology without *M. tuberculosis* infection.**

Representative fields of H&E-stained sections from the paraffin-embedded lungs that derived from water or antibiotics-treated mice with or without *M. tuberculosis* infection.

# Figure S4

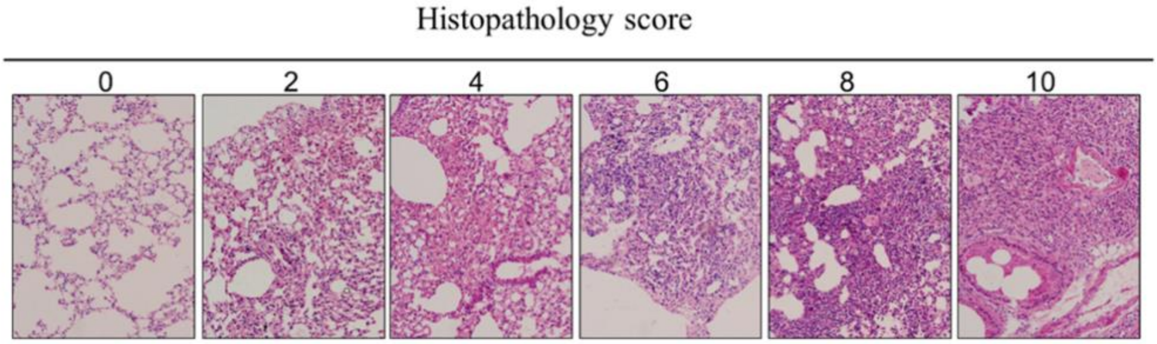


**Figure S4. Histopathology score scale.**

Representative fields of H&E-stained sections from the paraffin-embedded lungs of mice after *M. tuberculosis* infection. The histopathological parameters inflammatory lesions and granuloma formation were scored as absent(no inflammatory infiltrate in uninfected mice), minimal(little inflammatory infiltrate), slight(small-scale inflammatory infiltrate), moderate(necrosis and infiltration of inflammatory cells), marked(necrosis, large-scale inflammatory infiltrate and small-scale granuloma formation) or strong(observation of large-scale structure damages, necrosis and infiltration of inflammatory cells in the pulmonary compartments and granuloma formation), noted as 0-2, 2-2, 4-6, 6-8 ,and 8-10 , respectively. In this score the frequency as well as the severity of the lesions were incorporated. Granuloma formation was scored by estimating the occupied area of the lung section.The lungs of three animals were examined and the mean score of each of the four histological parameters was calculated.

# Figure S5

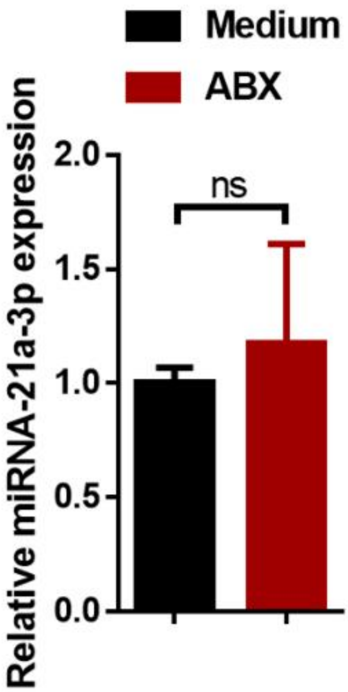


**Figure S5.** Relative expression of miR-21 expression in HEK 293T cells with or without antibiotics treatment was quantified by qPCR. Error bars indicated average values±SEM. ns: no statistical significance.

# Figure S6

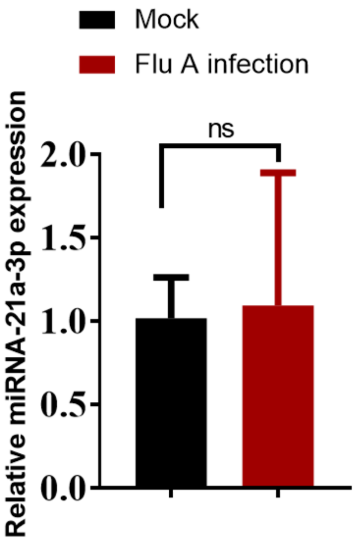


**Figure S6.** Relative expression of miR-21 in lung cells derived from mice in response to flu A infection or mock infection was quantified by qPCR. Error bars indicated average values±SEM. ns: no statistical significance.

# Figure S7

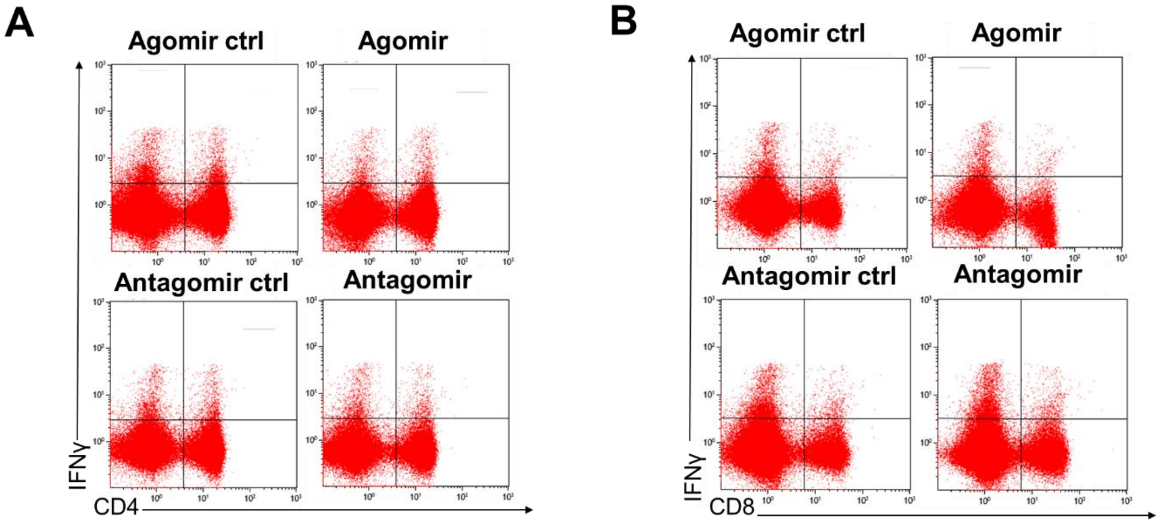


**Figure S7.** The CD4 versus IFN and CD8 versus IFN graphs without excluding CD4- or CD8- T cells related to Figure 5.

**Figure S8**

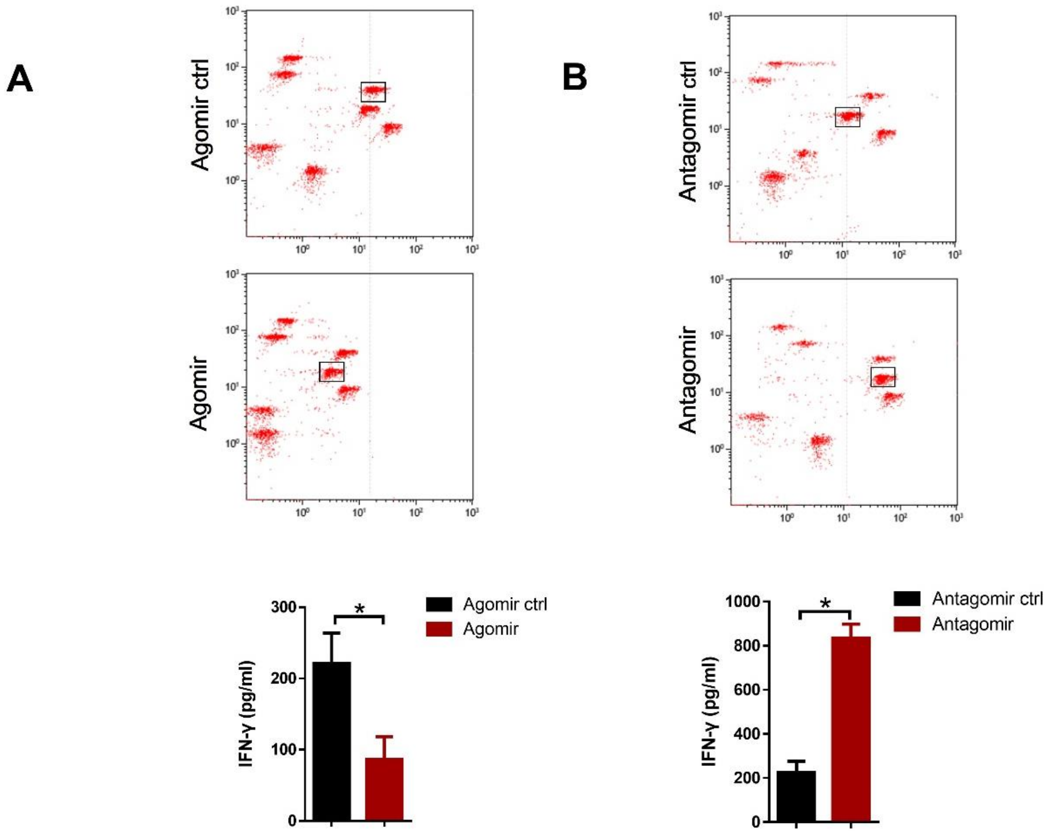


**Figure S8.** Cytokine expression profiles on CD4+ and CD8+ T cells by cytometric bead array kit (CBA). Cells from mice with agomir (n = 3) or antagomir (n = 3) and their respective controls(n=3) were stimulated with *Mtb* antigen ESAT-6 and incubated for 72 h. The levels of IFN-γ of (A) agomir and its control (B) antagomir and its control were calculated compared to standards. *$P < 0.05$, compared with the control group.
